# Validation of the web-based self-administered 24-h dietary recall myfood24-Germany: comparison with a weighed dietary record and biomarkers

**DOI:** 10.1007/s00394-021-02547-7

**Published:** 2021-05-11

**Authors:** Stefanie A. J. Koch, Johanna Conrad, Janet E. Cade, Leonie Weinhold, Ute Alexy, Ute Nöthlings

**Affiliations:** 1 Nutritional Epidemiology, Department of Nutrition and Food Sciences, University of Bonn, Bonn, Germany; 2 Science Department, German Nutrition Society, Bonn, Germany; 3 Nutritional Epidemiology Group, School of Food Science and Nutrition, University of Leeds, Leeds, UK; 4 Institute for Medical Biometry, Informatics and Epidemiology (IMBIE), University Hospital Bonn, Bonn, Germany

**Keywords:** Dietary assessment, Web-based 24-h dietary recall, Myfood24, Validation study

## Abstract

**Purpose:**

We aimed to validate myfood24-Germany, a web-based 24-h dietary recall (24HDR), by comparing its performance with a weighed dietary record (WDR) and biomarkers.

**Methods:**

97 adults (77% female) completed a 3-day WDR with a 24-h urine collection on day 3, followed by at least one 24HDR with myfood24-Germany (corresponding to day 3 of the WDR). Intake of energy and 32 nutrients assessed by myfood24-Germany and the WDR for the same day were compared (method comparison). Intakes of protein and potassium assessed by myfood24-Germany/WDR were compared with intake estimated from urinary biomarkers for protein and potassium (biomarker comparison).

**Results:**

In the method comparison, significant correlations were found for energy and all tested nutrients (range 0.45–0.87). There was no significant difference between both methods in the assessed mean energy and macronutrient intake. However, myfood24-Germany underestimated mean intake of 15 nutrients. In the biomarker comparison, protein intake reported by myfood24-Germany/WDR was on average 10%/8% lower than estimated by biomarker. There was no significant difference in mean potassium intake assessed by myfood24-Germany/WDR and biomarker. However, a shared bias in the assessment of potassium intake was observed for both instruments. Concordance correlation coefficients (*p*_c_) and weighted Kappa coefficients (*κ*) confirmed good agreement with the biomarker estimates for myfood24-Germany/WDR in case of protein (*p*_c_ = 0.58/0.66, *κ* = 0.51/0.53) and moderate agreement in case of potassium (*p*_c_ = 0.44/0.51; *κ* = 0.30/0.33).

**Conclusion:**

Our results suggest that myfood24-Germany is of comparable validity to traditional dietary assessment methods.

**Supplementary Information:**

The online version contains supplementary material available at 10.1007/s00394-021-02547-7.

## Introduction

Facing challenges in dietary assessment, a number of technology-based dietary assessment tools have been developed in the recent years [1, 2]. Web-based self-administered 24-h dietary recalls (24HDRs) are particularly promising to facilitate repeated short-term measurements, as proposed for the estimation of usual dietary intake in large-scale studies [3–5]. They offer potential advantages such as reduced time- and cost effort, lower burden for participants and researchers as well as increased quality and accuracy of data by process standardization [1, 6, 7]. They may also contribute to a more standardized dietary assessment across countries because they are adaptable for different populations [1, 8]. 

The fully automated online dietary assessment tool myfood24, first developed for use in the UK population, has been adapted for use in several other countries [7]. The development process of myfood24 as well as the adaption process for Germany has been described in detail elsewhere [7, 8]. Briefly described, to complete a 24HDR with myfood24, users enter their consumed foods on a website by searching food items in the underlying database. Features like portion size options, images and pop-up windows are implemented to guide the user through the self-administered recall. Within the adaptation process for the German version, a suitable database with German foods was developed. The underlying database includes 11,501 food and drink items (7,203 generic items, 4,298 branded products) and was built from the German Food Code and Nutrient Data Base (Bundeslebensmittelschlüssel (BLS) version 3.02) and the in‐house database of the Dortmund Nutritional and Anthropometric Longitudinally Designed (DONALD) study LEBTAB [9–11].

myfood24-UK has been validated against traditional 24HDRs (face-to-face interview and telephone interview) and biomarkers [12, 13]. The results indicated that myfood24-UK provides dietary intake measurement comparable to the more costly and time-consuming traditional 24HDRs. It has been shown to be feasible for use in different UK populations and study settings [12–17]. However, changing the underlying database means changing an essential part for its functionality, usability and accuracy [18, 19]. The different database underlying the adapted tool as well as the different target population may have an impact on validity [20, 21]. Therefore, the aim of this study was to validate myfood24-Germany by comparing its performance with a paper–pencil recorded WDR and urinary biomarkers.

## Materials and methods

### Recruitment and study design

Participants of the myfood24-Germany validation study were recruited at the campus of the University Bonn by oral advertisement and flyers as well as in the general German population by social media and press releases. Participants were eligible for participation if they were ≥ 18 years old, fluent in German, had regular high-speed internet access and a valid email address. Further, participants had to be stable in body-weight (not on a weight-loss diet), and willing to maintain their current dietary and activity behavior during the time of the study. In accordance with the quality criteria for dietary intake validation studies suggested by the EURopean micronutrient RECommendations Aligned Network of Excellence (EURRECA), we aimed to recruit 100 participants [22]. A number of 62 participants was needed to detect a mean difference in protein intake of 10% between methods (proc power in SAS®, test = paired t-test for ratios, alpha = 0.05, power = 0.8, CV = 0.3, Corr = 0.5).

On a first study visit, individuals were screened for eligibility. Participants were asked to keep a WDR for 3 consecutive days, collect a 24 h-urine on the third day and fill in four 24HDRs with myfood24-Germany. One day after keeping the WDR and collecting the 24 h-urine, participants visited the study center to hand in their WDRs and urines. During this second visit, they completed the first of four 24HDRs. That way, all participants completed one 24HDR and 1 day of WDR for the same day of consumption (first recalled day = third day of dietary recording). Further, body weight and height was measured and an online questionnaire was completed. Another three 24HDRs were completed independently at home within the following 4 weeks. After the final application of myfood24-Germany, participants were asked to answer an evaluation questionnaire online. Data on the evaluation has been published before [8]. An overview of the study design is shown in Online Resource 1.

### Dietary assessment and biomarkers

#### Weighed dietary record (reference method)

All participants received a paper-based form for the WDR as well as oral and written instructions on how to complete the WDR at their first study visit. Participants weighed and recorded all consumed foods and beverages, as well as leftovers on 3 consecutive days. When exact weighing was not possible, estimation of portion sizes was allowed but had to be clearly identified. Requested information included date, time and place of consumption as well as type, quantity and preparation of the consumed food. Participants were asked to describe the consumed foods with as much detail as possible (including e.g. brand name or fat content) or take product-photos (including the back-of-pack information on nutrients and ingredients). Self-made recipes had to be recorded by weighing and noting all ingredients and the quantity consumed after preparation. The WDRs were screened for inconsistencies at end of the second study visit. The WDRs were manually coded and independently reviewed at the study center of the DONALD study according to standard procedures [10, 23]. Within the DONALD study, WDRs are coded on a regular basis using the in-house database LEBTAB [11].

#### 24-h dietary recalls with myfood24-Germany (test method)

During the second study visit, participants handed in their WDR and 24-h urine, answered a questionnaire on health factors, sociodemographic data, dietary behavior and physical activity and completed the first 24HDR with myfood24-Germany. A study assistant introduced the main features of myfood24-Germany before participants started the 24HDR. In the following 4 weeks, participants received three invitation emails to complete further 24HDRs with myfood24-Germany at home. Participants who did not complete the 24HDRs within 1 day received a reminder e-mail. A maximum of three reminder emails per 24HDR were sent every 2 days. When a participant completed a 24HDR, a short feedback on macronutrient intake was displayed.

#### Collection and analyses of 24-h urine

At the first study visit, participants received a paper-based form for a urine protocol, storage and collection containers as well as detailed oral and written instructions on how to collect the 24-h urine sample. Urine collection was performed on the third day of the WDR. The first urine specimen in the morning was discarded and the time was recorded as the starting point for the urine protocol. In the following 24 h, all urine was collected and the respective time of urination was recorded. Participants also noted if a urine specimen could not be collected. Further, the intake of medication and supplements on the collection day was recorded. The 24-h urine protocols were screened for inconsistencies at the end of the second study visit.

The urine was directly forwarded to the laboratory where it underwent a routine check using a commercial test strip (Combur 5 Test® HC; Roche diagnostics GmbH, Mannheim, Germany). Further, weight and volume was determined before the samples were aliquoted and stored at ≤ -80 °C. Completeness of urine was verified by the urine protocols. Only samples with a total volume of > 500 ml and a self-reported collection time between 19.5 and 26 h as well as less than 4 h of reported non-collection were analysed (*n* = 89). Urine analyses were conducted at laboratories of the University Bonn. Nitrogen (N) in urine was measured in duplicate by Dumas method (rapid N exceed®, Elementar Analysensysteme, Langenselbold, Germany) [24], potassium (K) by atomic absorption spectroscopy (PerkinElmer® Atomic Absorption AAS 1100B, Perkin Elmer, Rodgau, Germany) and creatinine by Jaffé reaction (Beckman Creatinine Analyzer 2, Beckmann Coulter, Krefeld, Germany [25]). Protein intake according to urinary *N* was calculated under the assumptions that urine *N* is 80% of dietary *N* intake [26]. An overall nitrogen-to-protein conversion ratio of 6.25 was used. K intake according to urinary K was calculated under the assumptions that K excretion is 80% of dietary K intake [27, 28].

### Statistical analyses

For the present analyses, we compared intake values assessed for the same day of consumption, i.e., assessed by the first 24HDR, the third day of the WDR and the 24 h urine.

The automatic nutrient data output for the 24HDRs with myfood24-Germany was not edited for analyses except for two individuals. In both cases, participants entered a meal twice due to a technical problem. We deleted the second entered meal for the two known cases. The technical problem was reported to and solved by the myfood24 developers. In the DONALD study, supplements and medication are included in the coding process for the WDRs. For the present analyses, supplements and medication were excluded from the WDRs because they are not included in the automatic myfood24-Germany data output.

First, energy and nutrient intake assessed by myfood24-Germany and the WDR were compared (method comparison). Geometric mean intake values for energy and 32 nutrients were calculated. To confirm a linear relationship between measurements and to determine strength and direction of the association at individual level, spearman correlation coefficients for the crude intake values as well as for nutrient densities were calculated. To identify significant differences between both methods and describe their size and direction, the intake ratios (myfood24-Germany/WDR) and the 95% confidence limits were calculated using a paired t-test for ratios.

Second, intake of protein and potassium assessed by myfood24-Germany, WDR and biomarker were compared (biomarker comparison). In order to determine agreement at individual level and evaluate inter-method reliability, Lin’s concordance correlation coefficients (CCCs) were calculated. As CCCs are based on Pearson correlation, intake variables were log-transformed to improve normality. Further, a cross-quartile-classification was performed and weighted kappa coefficients were calculated based on raw intake variables.

To determine agreement at group level and describe size and direction of measurement errors, absolute and percent differences in intake between both assessment instruments (myfood24 and WDR) and biomarker were calculated. Mean differences as well as limits of agreements (LoA) were calculated for the log-transformed variables. Results were then back transformed representing the geometric mean ratios of intake between methods and the according LoA. The mean percent differences were derived from the obtained mean intake ratios. To graphically display the distribution of differences between the methods, enhanced Bland–Altman plots were generated. The plots combine a needle plot, showing the individual absolute differences between the two measurements, with a regression analysis for the differences between both measurements on the reference measurement. They provide a graphical identification of bias at group level [29].

The following sensitivity analyses were conducted for the biomarker comparison: (1) exclusion of participants with self-reported metabolic diseases, participants that reported taking medication or nutritional supplements at the day of urine collection and pregnant participants (*n* = 43); (2) exclusion of outliers in protein and potassium intake as assessed by myfood24-Germany, WDR and biomarker by identifying intake values smaller than Q1-1.5*IQR or bigger than Q3 + 1.5*IQR stratified by sex for each method of assessment (*n* = 15); (3) exclusion of potentially incomplete 24-h urine samples based on a creatinine index < 0.7 (*n* = 30) [30–32]. Creatinine index represents the ratio of expected to observed urine creatinine excretion within 24 h. It was calculated by the equation of Joossens & Geboers [31, 32].

All statistical analyses were performed using SAS procedures (version 9.4; Cary, NC, USA).

## Results

### Subject characteristics

**Fig. 1:**
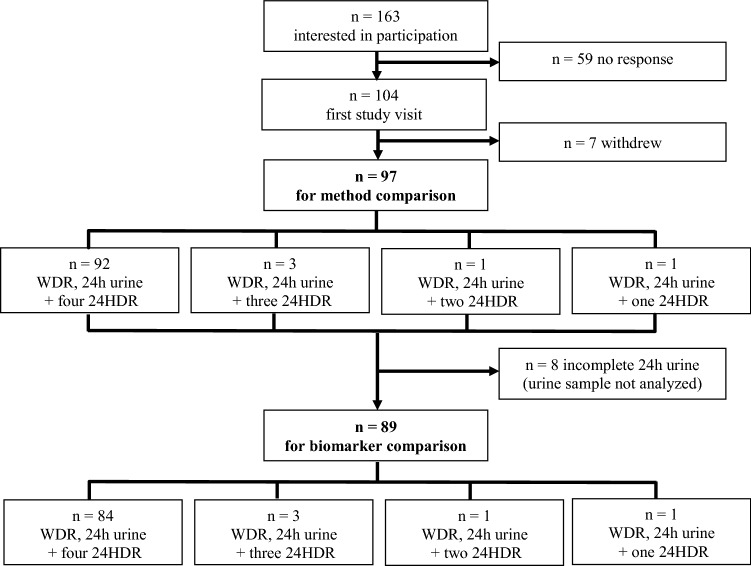
Flowchart illustrating the number of participants and completed three-day weighed dietary records (WDR), 24-h urine samples and 24-h recalls (24HDR)

Figure 1 illustrates the number of participants recruited and included in the statistical analyses. Ninety-seven participants were included in the study. All included participants kept a 3-day WDR, collected a 24-h urine on the third day of recording and completed a 24HDR with myfood24-Germany corresponding to day 3 of the WDR. Eighty-nine participants delivered a complete urine sample according to the criteria described above and were included in analyses for the biomarker comparison.

Table 1 shows the characteristics of the included participants. The age of participants ranged from 17 to 78 years, as one participant turned 18 during the course of the study. Median age was 30 years (Q1–Q3 25–61). The majority of participants were female (77%), had high educational status and experience in using computers. About 21% of the participants indicated having a nutritional or food scientific background while 11% reported having experience in completing a 24HDR. Mean BMI was in normal range for women and men. Only eight percent of participants reported being smokers. The majority of participants identified their current diet as omnivorous. Twelve participants reported being vegetarians and five reported being vegans.

**Table 1:** Characteristics of participants included in the myfood24-Germany validation study (*n* = 97)

	Men	Women
Participants, *n* (%)	22 (23)	75 (77)
Median age in years (Q1–Q3)	28 (24–54)	37 (25–62)
Mean BMI, kg/m^2^ (95% CI)	23 (22–24)	22 (22–23)
Smoker, n (% by group)	2 (9)	6 (8)
Education level, *n* (% by group)		
No school-leaving qualification	–	1 (1)
Trainee/Student	5 (23)	9 (12)
Vocational education	2 (9)	23 (31)
College/University degree	15 (68)	42 (56)
Type of diet, *n* (% by group)		
Omnivores	20 (91)	60 (80)
Vegetarians	1 (5)	11 (15)
Vegans	1 (5)	4 (5)
Nutrition professionals, n (% by group)	2 (9)	18 (24)
Conducted a 24HDR before, *n* (% by group)	–	11 (15)
Experience in using a computer/tablet, *n* (% by group)	19 (86)	56 (75)

Lower- (Q1) and upper-quartile (Q3), CI = confidence intervall

### Method comparison

The unadjusted mean intake values for energy and nutrients as assessed by myfood24-Germany and WDR as well as the mean ratio of intake (myfood24-Germany/WDR) and spearman correlation coefficients are shown in Table 2. There was no significant difference between both methods in energy intake and in intake of fat, carbohydrates and protein. Statistically significant correlations were found for all nutrients displayed, ranging from 0.45 for iodine intake to 0.87 for intake of cholesterol. However, for 15 of the investigated 32 nutrients, significant differences between the reported mean intakes by the 2 methods were found. The majority of those nutrients were vitamins (*n* = 9) as well as some micronutrients (*n* = 4) and additional values for SFA and total sugars. In case of significant differences between the assessed intake values, intake assessed by myfood24-Germany was lower than intake assessed by WDR. Overall, the statistically significant differences between both methods ranged from − 8% for total sugars to − 30% for vitamin B12.

**Table 2:** Mean energy and nutrient intake assessed by myfood24-Germany and a weighed dietary record (WDR) in *n* = 97 participants for the same day of consumption

Nutrient intake	myfood24 (*n* = 97 recalls)	WDR (*n* = 97 days)	Ratio of intake (myfood24/WDR)	Spearman Correlation^*^	
	Geo. mean (CV)	Geo. mean (CV)	Geo. mean (95% Cl)	Raw	Adjusted^a^
Energy (kcal)	1835 (0.31)	1909 (0.28)	0.96 (0.92, 1.01)	0.70	
Fat (g)	68.8 (0.59)	72.7 (0.53)	0.95 (0.89, 1.01)	0.78	0.76
SFA (g)	26.0 (0.71)	28.3 (0.63)	0.92 (0.85, 0.99)	0.81	0.76
MUFA (g)	23.4 (0.66)	25.0 (0.61)	0.93 (0.87, 1.01)	0.77	0.76
PUFA (g)	11.7 (0.74)	11.1 (0.68)	1.05 (0.97, 1.15)	0.77	0.78
Protein (g)	66.6 (0.48)	67.2 (0.42)	0.99 (0.94, 1.05)	0.81	0.80
Carbohydrate (g)	201.0 (0.37)	210.9 (0.35)	0.95 (0.90, 1.01)	0.66	0.77
Starch (g)	102.3 (0.65)	106.9 (0.57)	0.96 (0.88, 1.04)	0.71	0.77
Fibre (g)	22.9 (0.53)	23.9 (0.53)	0.96 (0.90, 1.02)	0.77	0.83
Total sugars (g)	77.2 (0.54)	84.4 (0.53)	0.92 (0.86, 0.98)	0.77	0.74
Cholesterol (mg)	134.3 (3.63)	125.8 (4.55)	1.07 (0.90, 1.27)	0.87	0.85
Alcohol (g)	0.7 (16.06)	0.7 (17.57)	1.03 (0.81, 1.32)	0.84	0.84
Sodium (g)	2.0 (0.61)	2.3 (0.60)	0.89 (0.81,0.98)	0.70	0.64
Potassium (g)	2.8 (0.40)	3.1 (0.38)	0.90 (0.86,0.96)	0.74	0.76
Calcium (g)	0.8 (0.54)	0.9 (0.54)	0.92 (0.86, 0.98)	0.79	0.70
Magnesium (g)	0.4 ( 0.44)	0.4 (0.43)	0.98 (0.93, 1.03)	0.75	0.80
Phosphorous (g)	1.2 (0.42)	1.3 (0.42)	0.95 (0.90, 1.01)	0.78	0.75
Iron (mg)	12.3 (0.49)	12.8 (0.48)	0.96 (0.90, 1.03)	0.75	0.71
Copper (mg)	1.7 (0.49)	2.0 (0.45)	0.85 (0.79, 0.91)	0.71	0.70
Zinc (mg)	9.7 (0.46)	10.2 (0.43)	0.95 (0.90, 1.01)	0.70	0.65
Iodine (ug)	81.2 (0.73)	91.2 (0.83)	0.89 (0.78, 1.02)	0.53	0.45
Retinol (Equ) (mg)	1.1 (0.94)	1.2 (0.89)	0.90 (0.82, 0.98)	0.83	0.81
β-carotene (mg)	3.2 (1.67)	4.1 (1.37)	0.77 (0.65, 0.92)	0.76	0.74
Vitamin D (ug)	1.6 (1.46)	1.8 (1.40)	0.93 (0.80, 1.09)	0.76	0.72
Vitamin E (mg)	11.7 (0.65)	12.9 (0.60)	0.91 (0.82, 1.00)	0.65	0.61
Thiamin (mg)	1.1 (0.52)	1.3 (0.50)	0.88 (0.81, 0.96)	0.66	0.66
Niacin (mg)	13.9 (0.55)	15.0 (0.50)	0.93 (0.86, 1.00)	0.72	0.77
Vitamin B6 (mg)	1.4 (0.55)	1.7 (0.45)	0.83 (0.77, 0.90)	0.74	0.72
Vitamin B12 (ug)	1.4 (2.18)	1.7 (1.48)	0.54 (0.46, 0.64)	0.80	0.73
Folate (Equ) (ug)	242.6 (0.54)	343.6 (0.44)	0.71 (0.65, 0.76)	0.61	0.61
Pantothenic acid (mg)	4.2 (0.54)	5.4 (0.45)	0.78 (0.73, 0.84)	0.68	0.71
Biotin (ug)	43.5 (0.58)	50.2 (0.53)	0.87 (0.80, 0.94)	0.70	0.64
Vitamin C (mg)	85.1 (1.09)	108.9 (0.98)	0.78 (0.69, 0.88)	0.78	0.79

Geo. mean = geometric mean, *CV* coefficient of variation

*p < 0.0001 for all displayed correlations

^a^Correlation for nutrient densities (nutrient intake per 1000 kcal), a constant was added to each individual intake value when the calculation of geometric mean was not possible due to intake value of zero and subtracted from the result for absolute numbers (alcohol, vitamin D, vitamin B12)

### Biomarker comparison

Mean intake values for protein and potassium as assessed by myfood24-Germany, WDR and biomarker are shown in Table 3. Table 4 shows the absolute and percentage differences in protein and potassium intake assessed by myfood24-Germany, WDR and biomarkers as well as the 95% LoA. Protein intake reported by myfood24-Germany and WDR was on average 10% (LoA − 53% to + 72%) and 8% (LoA − 46% to + 56%) lower than the protein intake estimated by biomarker. No significant difference in mean protein intake assessed by myfood24-Germany and WDR was found (LoA − 43% to + 67%). There was no significant difference in mean potassium intake assessed by myfood24-Germany or WDR and biomarker (LoA − 57% to + 98% and − 49% to + 103%, respectively). Potassium intake assessed by myfood24-Germany was on average 10% lower than potassium intake assessed by WDR (LoA: -45% to + 47%). For all six comparisons, more than 95% of the individual intake values fell within the calculated LoA (data not shown).

**Table 3:** Mean intake of protein and potassium assessed by myfood24-Germany, weighed dietary record and urinary biomarker (*n* = 89)

Assessment method	Protein intake	Potassium intake
	Geometric mean (95% CI)	Geometric mean (95% CI)
myfood24	66.8 (60.9, 73.3)	2.8 (2.6, 3.1)
WDR	68.4 (63.1, 74.1)	3.1 (2.9, 3.4)
biomarker	74.4 (70.0, 79.1)	3.1 (2.9, 3.3)

myfood24 = myfood24-Germany, *WDR* weighed dietary record, *Cl* confidence limits for geometric mean

**Table 4:** Absolute and percentage differences in nutrient intake and limits of agreement (LoA) for assessment instruments and biomarker (*n* = 89)

	Differences in nutrient intake			% LoA^a^
	Absolute (g)		Percentages (%)^a^	
	Mean (95%CI)	Median (95%Cl)	Mean (95%CI)	
Protein				
myfood24—biomarker	− 3.86 (− 10.25, 2.53)	− 7.89 (− 13.50, − 1.18)	− 10% (− 16%, − 4%)	− 53% to + 72%
WDR -biomarker	− 4.30 (− 8.69, 0.10)	− 5.15 (− 8.80, − 0.10)	− 8% (− 13%, − 3%)	− 46% to + 56%
myfood24—WDR	0.44 (− 5.25, 6.13)	− 1.27 (− 6.29, 2.24)	− 2% (− 8%, + 3%)	− 43% to + 67%
Potassium				
myfood24—biomarker	− 0.24 (− 0.53, 0.06)	− 0.27 (− 0.48, 0.12)	− 8% (− 15%, 0%)	− 57% to + 98%
WDR -biomarker	0.04 (− 0.24, 0.32)	0.06 (− 0.17, 0.34)	+ 2% (− 5%, + 10%)	− 49% to + 103%
myfood24—WDR	− 0.28 (− 0.45, − 0.11)	− 0.23 (− 0.36, − 0.15)	− 10% (− 14%, − 5%)	− 45% to + 47%

*LoA* Limits of agreement, *WDR* Weighed Dietary Record

^a^% difference in means and limits of agreement relate to geometric means of intake ratios because of log transformation and are presented as % differences

**Fig. 2:**
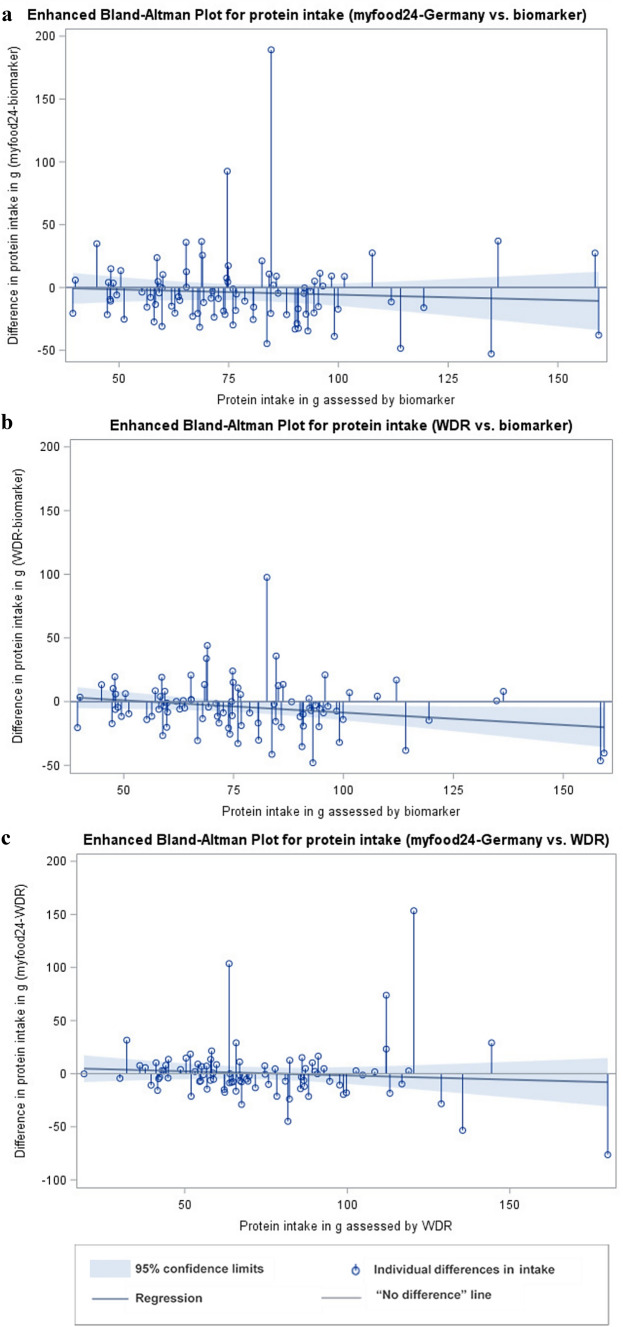
Enhanced Bland–Altman Plots for protein intake. (**a**) Comparison between myfood24-Germany and biomarker estimation, (**b**) Comparison between weighed dietary record (WDR) and biomarker estimation, (**c**) Comparison between myfood24-Germany and WDR

**Fig. 3:**
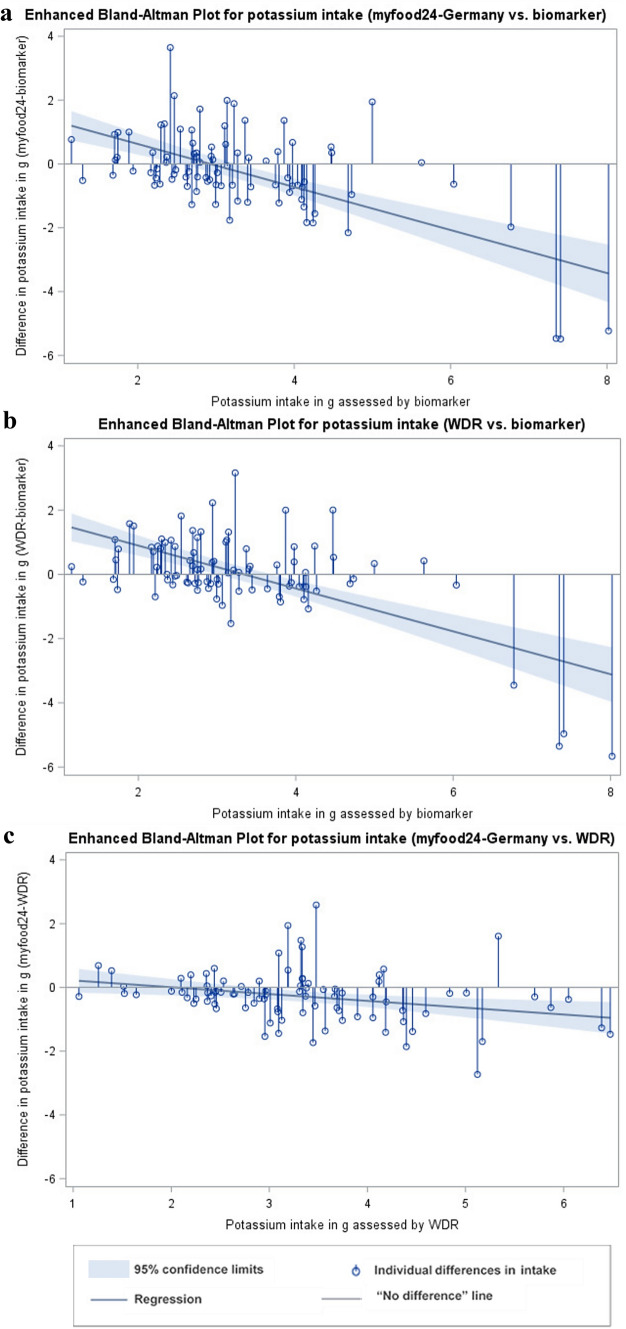
Enhanced Bland–Altman Plots for potassium intake. (**a**) Comparison between myfood24-Germany and biomarker estimation, (**b**) Comparison between weighed dietary record (WDR) and biomarker estimation, (**c**) Comparison between myfood24-Germany and WDR

The enhanced Bland–Altman plots for protein and potassium are shown in Figs. 2 and 3. There was no significant bias present in the assessment of protein intake when comparing all three assessment methods (myfood24-Germany, WDR and biomarker). For the majority of individuals protein intake assessed by myfood24-Germany and WDR was lower than estimated by biomarker (for 64% and 62%, respectively). For 57% of individuals, protein intake was lower assessed by myfood24-Germany compared to the WDR.

For potassium intake, the enhanced Bland–Altman Plots looked similar for both assessment instruments, when compared to the biomarker. There was a bias present in the assessment. The needle plot showed a tendency to overestimate potassium intake at low range of intake values and underestimate potassium intake at high range of intake values. For the majority of individuals, potassium intake was lower assessed by myfood24-Germany (66%) and higher assessed by WDR (54%) when compared to the biomarker. The plot for the comparison between myfood24-Germany and the WDR showed that the degree of differences was higher in the middle and upper range of intake values. Further, myfood24-Germany tended to underestimate potassium intake at high range of intake values compared to the WDR.

**Fig. 4:**
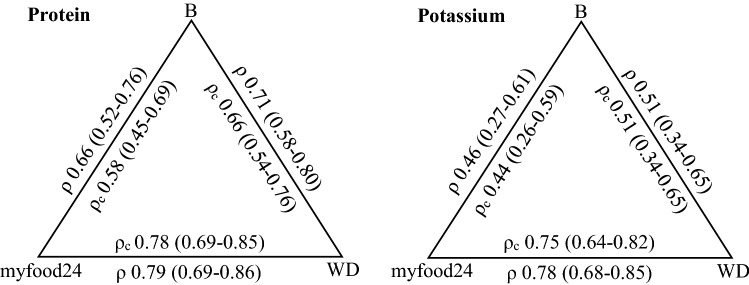
Pearson’s Correlation Coefficients (ρ (95% CI)) and Lin’s concordance correlation coefficients (ρ_c_ (95% CI)) for intake of protein and potassium according to urinary biomarkers nitrogen and potassium (B), myfood24-Germany (myfood24) and weighed dietary record (WDR). Measurements were based on one 24-h dietary recall, 1 day of WDR and one 24-h urine completed for the same day by *n* = 89 participants. Intake variables were log transformed to improve normal distribution

Pearson correlation coefficients and CCCs are shown in Fig. 4. Statistically significant positive correlations were found for all six comparisons. For protein intake, the CCC between myfood24-Germany and the biomarker was lower but of similar magnitude as the CCC between the WDR and the biomarker (0.58 (0.45–0.69) and 0.66 (0.54–0.76), respectively). CCCs for potassium intake were overall lower than for protein intake but showed a similar relation. The CCC between myfood24-Germany and the biomarker was lower but of similar magnitude as the CCC between the WDR and the biomarker (0.44 (0.26–0.59) and 0.51 (0.34–0.65), respectively). For both nutrients, highest agreement according to CCC was found for myfood24-Germany and the WDR (protein: 0.78 (0.69–0.85), potassium: 0.75 (0.64–0.82)).

Results of the cross-classification are shown in Table 5. Compared to the biomarker, a similar proportion of participants was classified into the different quartiles of protein and potassium intake by both methods (myfood24-Germany and WDR). The percentage of cases classified into the same or an adjacent quartile was 90% and 80% for protein and potassium intake, respectively. Weighted kappa coefficient was 0.51/0.30 for myfood24-Germany and 0.53/0.33 for WDR in case of protein and potassium, respectively. In concordance with the CCCs, highest agreement was found between both self-report methods. The percentage of cases classified into the same quartile by myfood24-Germany and WDR was > 50%. Less than 10% were classified two quartiles apart. Weighted kappa coefficient was 0.60 and 0.62 for protein and potassium, respectively.

**Table 5:** Cross-classification of quartiles of nutrient intake as assessed by myfood24-Germany, weighed food record and urinary biomarker in the same day (*n* = 89)

	Same n (%)	Same and adjacent *n* (%)	Two apart *n* (%)	Three apart *n* (%)	κ (95% Cl)
Protein				–	
myfood24 & biomarker	44 (49.4)	80 (89.9)	9 (10.1)	–	0.51 (0.39–0.63)
wdr & biomarker	47 (52.8)	80 (89.9)	8 (9.0)	1 (1.1)	0.53 (0.41–0.65)
myfood24 & wdr	51 (57.3)	83 (93.3)	6 (6.7)	–	0.60 (0.49–0.72)
Potassium					
myfood24 & biomarker	32 (36.0)	71 (79.8)	15 (16.9)	3 (3.4)	0.30 (0.15–0.44)
wdr & biomarker	34 (38.2)	73 (82.0)	13 (14.6)	3 (3.4)	0.33 (0.19–0.47)
myfood24 & wdr	55 (61.8)	81 (91.0)	8 (9.0)	–	0.62 (0.51–0.74)

*Κ*  weighted Kappa coefficient, Same: number and percentage of cases cross-classified into the same quartile. Same and adjacent: number and percentage of cases cross-classified into the same or an adjacent quartile. Two apart: number and percentage of cases cross-classified 2 quartiles apart. Three apart: number and percentage of cases cross-classified 3 quartiles apart

The results of the sensitivity analyses did not substantially differ from the results described here in the main analysis.

## Discussion

To the author’s knowledge, myfood24-Germany is the first self-administered web-based dietary assessment tool available for Germany. In comparison to urinary biomarkers, myfood24-Germany performed similarly well to the traditional paper–pencil recorded WDR. The new instrument showed good agreement with the WDR in short-term assessment of energy and nutrient intake. The biomarker comparison confirmed the good agreement between myfood24-Germany and the WDR and provided additional insights into the extent and type of the associated measurement errors for both methods.

The spearman correlation coefficients confirmed moderate to very strong linear relationships between intake values assessed by myfood24-Germany and the WDR for all tested nutrients. To our knowledge, no other study compared a web-based 24HDR with a WDR but rather traditional 24HDRs. The magnitude and range of the observed correlations was comparable to other studies, where a web-based 24HDR was compared with a traditional 24HDR for one time point [33, 34]. Timon et al. compared a web-based 24HDR to a semi-weighed dietary record [35]. The reported correlations for nutrient intake were overall weaker than in the present study (range 0.32–0.75). However, they compared intake values assessed by three non-consecutive 24HDRs and 4 consecutive days of dietary recording completed at different time points. Weaker correlations may therefore be explained by day-to-day variation in dietary intake. Similar to Timon’s results, a range of correlations between 0.34 and 0.75 was found for myfood24-Germany and WDR, when we compared mean values of all completed 24HDRs (*n* = 380 recalls) and WDRs (*n* = 291 days) in the present study (Online Resource 2). Since no inferences on agreement can be made based on correlation coefficients, we also calculated the ratios between both methods for intake of energy and a range of nutrients. There were no significant differences between myfood24-Germany and WDR in the assessment of energy and macronutrients. Significant differences between both methods were found for a range of micronutrients, where intake assessed with myfood24-Germany was lower than intake assessed by WDR. One possible explanation might be the error in portion size estimation in the 24HDR compared to weighing in the WDR. The estimation of portion sizes is a major challenge for study participants. Additionally, participants might not have remembered all consumed foods during the 24HDR. These problems are well known for all retrospective methods of dietary assessment [36, 37]. Underreporting has also consistently been found for macro- and micronutrients in studies using traditional or web-based 24HDRs [5, 38, 39]. A rarely investigated problem emerging with self-administration might also affect the agreement between myfood24-Germany and the WDR. Participants are asked to identify and chose correct items representing the consumed foods directly from the underlying database to complete a 24HDR with myfood24-Germany. Comparable to most other web-based 24HDRs, there is no option to add foods that were missing or not found to facilitate the automated coding [1]. Instead, participants are asked to choose suitable substitutes, which may lead to differences in single nutrient values for individuals [8, 40, 41]. This might particularly affect micronutrients when fortified products, such as iodized salt, are reported incorrectly. Nevertheless, concordance correlation coefficients, cross-classification and weighted Kappa coefficients indicated good agreement between myfood24-Germany and WDR for the assessment of protein and potassium intake.

The biomarker comparison revealed that both tools, myfood24-Germany and WDR, underestimated protein intake on a similar and acceptable level [42]. However, percent difference was slightly higher and LoA wider for myfood24-Germany, indicating a little less accuracy and precision in the myfood24-Germany measurements. This was also reflected in the CCCs and the cross-classification. These findings are in line with a pooled analysis of validation studies of dietary self-report instruments using recovery biomarkers conducted by Freedman et al. [39]. They found that protein intake was underreported with 24HDRs by an average of 5% (range across studies: + 20% to − 21%). Further, results of a study comparing an automated self-administered 24HDR, a 4-day dietary record and a FFQ against biomarkers showed that protein intake was underestimated by all three instruments, whereby the 24HDRs performed as well as the dietary record and both performed better than the FFQs [5].

Even though there was no significant percentage difference in potassium intake found when myfood24-Germany and WDR were compared to the biomarker, CCCs and cross-classification indicated only moderate agreement with the biomarker for both tools. The enhanced Bland–Altman plot suggests that intake was overestimated at low range and underestimated at high range of intake by both self-report tools. This was partly caused by very high intake values of four individuals estimated by biomarker excretion (> 6 g). Further, the observed relation between the differences and the magnitude of intake might be induced by the chosen method of analysis [43]. Still, a shared measurement error in the assessment of potassium intake cannot be excluded as an influencing factor for the agreement between myfood24-Germany and WDR. In self-report dietary assessment it is common that participants with higher true intake tend to under-report and those with lower true intake tend to over-report [44]. This common phenomenon of regression towards the mean, would also lead to higher differences between intake values assessed by self-report tools and true intake in lower and higher range of intake values as seen for potassium in the present study. Another possible source for a shared measurement error between myfood24-Germany and WDR might be the underlying databases. The underlying database of myfood24-Germany includes a high number of branded food items from LEBTAB (around 40% [8]), the database used for the coding of the WDRs in the present study. Further, nutrient values for staple foods in LEBTAB were mainly obtained from the BLS (93.2%), which was also the source for generic items in myfood24-Germany [8]. Thus, there is a large overlap between the underlying database of myfood24-Germany and the WDRs.

The results of the present study suggest that myfood24-Germany provides short-term intake estimates for energy and a range of nutrients that are comparable to a WDR. Both methods showed a similar extent of measurement error compared to biomarkers. These findings are in line with other studies where web-based 24HDRs were compared with traditional dietary assessment instruments and/or biomarkers [5, 13, 45]. For example, the web-based 24HDR ASA24 performed similarly to estimated dietary records, and fairly well compared to biomarkers in the assessment of energy, protein, potassium and sodium [5]. Consistent with our results, protein intake was underestimated by both instruments compared to biomarker. Lassale et al. compared a web-based dietary record tool to urinary biomarkers for protein, potassium and sodium [45]. They found that protein was underreported by around 14% by both sexes, while potassium was underreported by around 4% by women only. Correlation coefficients for intake values of protein and potassium assessed for the same day by the web-based dietary record and biomarkers were slightly higher but in the same range as found for myfood24-Germany in the present study. Further, myfood24-UK was compared to a traditional interviewer-led 24HDR in the assessment of long-term intake in a validation study that also included biomarkers and found similar results [13].

Clearly, web-based dietary assessment tools, including myfood24-Germany, do not yet overcome measurement errors and biases that are associated with traditional dietary assessment instruments. However, they seem to offer intake estimates of comparable validity. At the same time, they are more time- and cost-efficient and thereby more feasible in large-scale epidemiological studies, where usual dietary intake is commonly the exposure of interest. Still, FFQs are often preferred for the assessment of usual dietary intake, although the evidence suggests that repeated 24-h recalls, at best combined with a FFQ, are better suited for that purpose [4, 5, 39]. One reason might be the limited availability of web-based 24HDRs in some countries.

Our study had some strengths and limitations. With the WDR, we used a reference method for our method comparison known to be most precise and accurate among the available self-report dietary assessment instruments. Another major strength is the use of objective recovery biomarkers to partly verify the results of our method comparison and estimate the extent of measurement error associated with the test and reference method. A sufficient number of statistical tests were performed to provide insights into different facets of validity [42]. As for the present study, intake values assessed for the same day of consumption were compared, intra-individual variation of diet had no impact on the results. However, this also might have had a positive influence on the determined measures of validity because participants first weighed and recorded all consumed foods for a day and were asked to recall all consumed foods for the same day while completing the 24HDR with myfood24-Germany 1 day later. Hence, participants had better memory of the consumed foods than in an anticipated 24HDR situation. No PABA was used to assess the completeness of the 24-urine samples. However, comprehensive urine protocols and additional criteria were used to ensure accuracy of the urine collection. The results from the biomarker comparison are limited to protein and potassium and cannot be transferred to energy and other macro- or micronutrients. Due to the limited number of collected 24-h urine samples, the present validation relates only to short-term intake. However, most participants (*n* = 92) completed four 24HDRs and the user-evaluation confirmed that myfood24 is feasible for repeated short‐term application [8]. Due to convenience sampling, the study population was not representative of the general German population, which limits the generalizability of our results. However, adult participants from different age groups were represented. Future studies will give rise to more knowledge about usability and validity of myfood24-Germany for diverse study populations. Generally, participants should be introduced to the functions and features of myfood24‐Germany to avoid usability problems and increase the accuracy of the self-administered food entries [8].

## Conclusion

myfood24-Germany provides short-term intake estimates for energy and a range of nutrients that are comparable to a WDR. Both methods showed a similar extent of measurement error compared to biomarkers. Our results suggest that myfood24-Germany is of comparable validity to more costly and time-consuming traditional dietary assessment methods.

## Supplementary Information

Below is the link to the electronic supplementary material.

Supplementary file1 (PDF 135 kb)

## Data Availability

Upon request to epi@uni-bonn.de. Requests to use myfood24 should be made to enquiries@myfood24.org.
