# Supplementary material for: Validation of the web-based self-administered 24-h dietary recall myfood24-Germany: comparison with a weighed dietary record and biomarkers
Source: Eur J Nutr. 2021 May 11;60(7):4069–82. doi: 10.1007/s00394-021-02547-7 (PMC8437850; doi:10.1007/s00394-021-02547-7)
Supplement: Supplementary file 1 — Supplementary file1 (PDF 135 kb) [file 394_2021_2547_MOESM1_ESM.pdf]

Validation of the Web-Based Self-Administered 24-h Dietary Recall myfood24-Germany: comparison with a weighed dietary record and biomarkers

Stefanie A. J. Koch[a*] (ORCID 0000-0001-7399-7188), Johanna Conrad[a, b] (ORCID 0000-0001-5047-1669), Janet E. Cade[c] (ORCID 0000-0003-3421-0121), Leonie Weinhold[d] (ORCID 0000-0002-9194-9427), Ute Alexy[a] (ORCRID 0000-0002-1488-5175), Ute Nöthlings[a] (ORCID 0000-0002-5789-2252)

[a] Nutritional Epidemiology, Department of Nutrition and Food Sciences, University of Bonn, Germany, [b] Science Department, German Nutrition Society, Bonn, Germany [c] Nutritional Epidemiology Group, School of Food Science and Nutrition, University of Leeds, UK, [d] Institute for Medical Biometry, Informatics and Epidemiology (IMBIE), University Hospital Bonn, Germany

* Corresponding author:	E-mail address:	s.koch@uni-bonn.de
	Phone number:	+49 (0) 228 73 69861

Supplementary Material

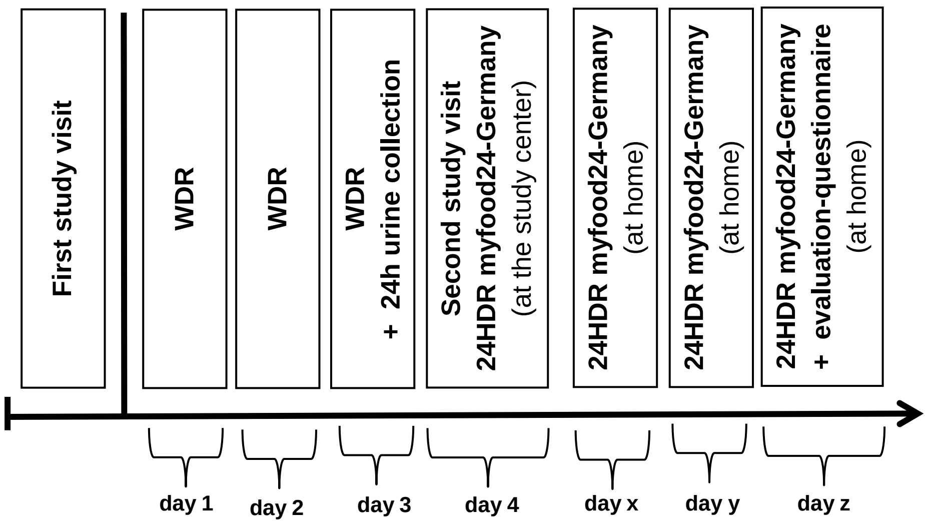

Online Resource 1 Study design overview (WDR=Weighed Dietary Record, 24HDR=24-hour Recall)

Supplementary Material

Online Resource 2 Mean energy and nutrient intake assessed by 380 24-hour recalls with myfood24-Germany and 291 days of weighed dietary recording (WDR) in n=97 participants.

	**myfood24 (n=380 recalls)**	**WDR (n=291 days)**	**Ratio of intake (myfood24/WDR)**	**Spearman Correlation[*]**	
**Nutrient intake**	**Geo. mean (CV)**	**Geo. mean (CV)**	**Geo. mean (95% Cl)**	**Raw**	**Adjusted[a]**
Energy (kcal)	1850 (0.35)	1894 (0.32)	0.99 (0.95, 1.03)	0.65	
Fat (g)	71.3 (0.56)	71.2 (0.53)	1.02 (0.96, 1.09)	0.61	0.63
SFA (g)	27.5 (0.67)	27.4 (0.65)	1.03 (0.95, 1.10)	0.64	0.62
MUFA (g)	24.3 (0.63)	24.3 (0.58)	1.03 (0.96, 1.11)	0.55	0.53
PUFA (g)	11.5 (0.72)	11.1 (0.69)	1.08 (1.00, 1.17)	0.60	0.61
Protein (g)	65.5 (0.46)	66.1 (0.43)	1.02 (0.96, 1.07)	0.72	0.54
Carbohydrate (g)	203.0 (0.38)	213.1 (0.40)	0.96 (0.92, 1.01)	0.67	0.74
Starch (g)	103.0 (0.73)	106.8 (0.62)	0.98 (0.92, 1.05)	0.73	0.75
Fibre (g)	21.8 (0.54)	23.4 (0.55)	0.94 (0.87, 1.00)	0.64	0.68
Total sugars (g)	77.4 (0.60)	86.4 (0.59)	0.92 (0.86, 0.98)	0.65	0.69
Cholesterol (mg)	150.5 (2.99)	131.8 (3.26)	1.23 (1.02, 1.49)	0.62	0.60
Alcohol (g)	0.8 (17.04)	0.7 (16.99)	1.36 (0.94, 1.98)	0.62	0.62
Sodium (g)	2.1 (0.59)	2.4 (0.56)	0.90 (0.84, 0.97)	0.52	0.42
Potassium (g)	2.7 (0.43)	3.0 (0.40)	0.91 (0.87, 0.96)	0.59	0.60
Calcium (g)	0.8 (0.53)	0.8 (0.56)	0.94 (0.89, 1.00)	0.72	0.60
Magnesium (g)	0.3 (0.45)	0.4 (0.42)	0.98 (0.93, 1.03)	0.75	0.70
Phosphorous (g)	1.2 (0.42)	1.2 (0.42)	0.97 (0.93, 1.02)	0.75	0.56
Iron (mg)	12.0 (0.50)	12.6 (0.43)	0.98 (0.93, 1.03)	0.72	0.63
Copper (mg)	1.6 (0.52)	1.9 (0.46)	0.88 (0.83, 0.95)	0.66	0.60
Zinc (mg)	9.6 (0.46)	9.9 (0.40)	0.99 (0.94, 1.04)	0.65	0.43
Iodine (ug)	79.01 (0.68)	91.1 (0.84)	0.85 (0.76, 0.94)	0.41	0.34
Retinol (Equ) (mg)	1.0 (0.88)	1.2 (0.96)	0.84 (0.75, 0.94)	0.45	0.39
β-carotene (mg)	2.8 (1.59)	4.0 (1.52)	0.74 (0.64, 0.86)	0.53	0.49
Vitamin D (ug)	1.6 (1.47)	1.6 (1.34)	1.16 (1.01, 1.33)	0.53	0.54
Vitamin E (mg)	11.4 (0.63)	13.0 (0.69)	0.88 (0.81, 0.96)	0.47	0.38

Thiamin (mg)	1.1 (0.51)	1.3 (0.49)	0.89 (0.82, 0.95)	0.50	0.45
Niacin (mg)	13.1 (0.60)	14.7 (0.55)	0.92 (0.86, 1.00)	0.51	0.46
Vitamin B6 (mg)	1.3 (0.59)	1.7 (0.53)	0.82 (0.77, 0.89)	0.49	0.47
Vitamin B12 (ug)	1.5 (2.21)	2.7 (1.41)	0.65 (0.57, 0.74)	0.72	0.68
Folate (Equ) (ug)	233.4 (0.54)	334.7 (0.52)	0.70 (0.65, 0.75)	0.53	0.50
Pantothenic acid (mg)	4.0 (0.55)	5.3 (0.46)	0.78 (0.73, 0.83)	0.59	0.54
Biotin (ug)	42.4 (0.58)	50.7 (0.55)	0.86 (0.80, 0.92)	0.65	0.64
Vitamin C (mg)	78.8 (1.20)	103.9 (1.06)	0.79 (0.70, 0.89)	0.58	0.60

*Geo. mean=geometric mean, CV=coefficient of variation, * p<.0001 for all displayed correlations, [a] correlation for nutrient densities (nutrient intake per 1000kcal), a constant was added to each individual intake value when the calculation of geometric mean was not possible due to intake values of zero and subtracted from the result for absolute numbers (alcohol, cholesterol, vitamin D, vitamin B12, vitamin C)*
